# *In Vitro* Delivery and Controlled Release of Doxorubicin for Targeting Osteosarcoma Bone Cancer

**DOI:** 10.3390/molecules180910580

**Published:** 2013-08-30

**Authors:** Shafiu Abdullahi Kamba, Maznah Ismail, Samer Hasan Hussein-Al-Ali, Tengku Azmi Tengku Ibrahim, Zuki Abu Bakar Zakaria

**Affiliations:** 1 Laboratory of Molecular Biomedicine, Institute of Bioscience, University Putra Malaysia, UPM 43400, Serdang, Malaysia; E-Mails: shafiuabdullahi@yahoo.com (S.A.K.); maznah@medic.upm.edu.my (M.I.); sameralali72@yahoo.com (S.H.H.-A.-A.); 2 Faculty of Veterinary Medicine, University Putra Malaysia, UPM 43400, Serdang, Malaysia; E-Mail: tengku@vet.upm.edu.my

**Keywords:** drug delivery, calcium carbonate, nanocrystals, doxorubicin, osteosarcoma

## Abstract

Drug delivery systems are designed to achieve drug therapeutic index and enhance the efficacy of controlled drug release targeting with specificity and selectivity by successful delivery of therapeutic agents at the desired sites without affecting the non-diseased neighbouring cells or tissues. In this research, we developed and demonstrated a bio-based calcium carbonate nanocrystals carrier that can be loaded with anticancer drug and selectively deliver it to cancer cells with high specificity by achieving the effective osteosarcoma cancer cell death without inducing specific toxicity. The results showed pH sensitivity of the controlled release characteristics of the drug at normal physiological pH 7.4 with approximately 80% released within 1,200 min but when exposed pH 4.8 the corresponding 80% was released in 50 min. This study showed that the DOX-loaded CaCO_3_ nanocrystals have promising applications in delivery of anticancer drugs.

## 1. Introduction

Development of controlled delivery systems with less or no toxicity is the main objective of researchers in medicine, pharmaceutical scientists, medicinal chemists and other health related disciplines. The search for alternative treatments like drug delivery carriers is necessary since most chemotherapeutic drugs, especially anti cancer drugs, are considerably toxic to normal cells or lack specificity and selectivity which prevent the use of high dosages in the treatment [1]. Currently doxorubicin is one of the effective and broad spectrum anti cancer drugs used in the treatment of different solid tumours [2,3], but the use of doxorubicin in bone sarcoma treatment is limited because it was reported that achieving a chemotherapeutic dosage through systemic delivery is difficult due to the like of specificity and selectivity of conventional delivery systems [3]. This resulted in a narrow therapeutic index and significant increases in the high dose distribution to healthy normal tissues [3,4] and among the major side effects of doxorubicin which limit the clinical use of the drug are cardiotoxicity, myelosuppression and mucositis [4]. Recently in seeking an effective delivery system, attention have been focused on the use of nanomedicine or nanotherapuetic delivery systems which have numerous advantages and offer promising results in solving the major drawback in conventional delivery systeme. Such advantages are achieved through increase of drug solubility, specific tumor targeting, enhanced accumulation in tumor tissues and tumor cells, decreased systemic toxicity and increased maximum tolerate dosage [1]. To confront such problems and increase the effectiveness of Dox in sarcoma treatment, it was recently reported that delivery through calcium phosphate-reinforced polymer nanoparticles and also in combinations with inorganic phosphate have improved the therapeutic index and selectivity of Dox in the treatment of osteosarcoma [5,6]. Nanodrugs can selectively accumulate in tumors through a passive targeting mechanism known as the enhanced permeability and retention (EPR) effect [1].

The system used bio-based calcium carbonate nanocrystals of aragonite polymorph from cockle shells (*Anadara granosa*) in which the primary composition is approximately 98%–99% calcium carbonate [7]. This research was the first to be reported in the area delivery system. To the best of our knowledge no report or research was done using the same material in this regard. Herein, the research focus on the use of calcium carbonate as the main sources of calcium and carbonate ions respectively which is in contrary to the most of reported literatures where the researchers described the use of chemical reactions such as co-precipitation or double decomposition methods whereby salts of calcium and carbonate ions such as reactions between CaCl_2_ and Na_2_CO_3_, CaCl_2_ and NH_4_CO_3_ or Ca(NO_2_)_3_ and Na_2_CO_3_, which act as the precursor for the synthesis of calcium carbonate nanocrystals [7].

## 2. Results and Discussion

### 2.1. Transmission Electron Microscope (TEM) and Field Emission Scanning Electron Microscope (FESEM) Remains the Most Important Instrument Used in Nanoparticles Characterisation, Microscopy is The Only Method in Which the Individual Particle Size, and Morphology Are Directly Observed and Measured

Figure 1a,b are micrograph images by transmission electron microscope (TEM) and field emission scanning electron microscope (FESEM) for the synthesized calcium carbonate nanocrystals loaded with doxorubicin drug (CaCO_3_/Dox). The demonstrated micrographs in Figure 1a,b revealed a rod shape image of the synthesized CaCO_3_/Dox with an average diameter of the particles within 100 nm. The FESEM calcium carbonate particles image indicates a porous nature for the surface of the particles whereby the CaCO_3_ may encapsulate the drugs during physical interclation of the drug. The idea of our present research is to specifically use rod-shaped calcium carbonate aragonite polymorph in the delivery of anticancer drugs. The rod shape image shown in Figure 1a,b after loading of the drug is maintained, therefore as described by other researchers, rod shape nanocariers constitute unique characteristic, more selective and effective cancer delivery systems compared with the spherical shape of the same material [8].

**Figure 1:**
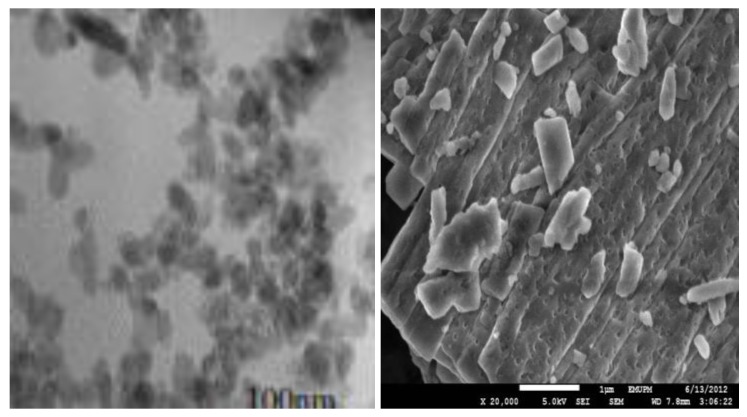
(**a**) TEM micrograph of CaCO_3_/Dox nanocrystal and (**b**) FESEM micrograph of CaCO_3_/Dox.

### 2.2. X-Ray Diffraction (XRD)

X-ray diffraction (XRD) is a strong analytical tool used to measure the crystalline content of materials by identifying the crystalline phases present and also for the quantification of mixtures in a given sample. The XRD spectra of pure doxorubicin, nude CaCO_3_ nanocrystals and CaCO_3_/Dox are presented in Figure 2. Strong crystallisations were observed in both the nude calcium carbonate and doxorubicin-loaded calcium carbonate nanocrystal spectra as indicated in Figure 2. The prominent peaks in nude CaCO_3_ nanocrystal and CaCO_3_/Dox are within 2θ = 26.5° and 27° with another 2θ = 33.3° and 34.8°, the XRD data suggest the crystal nature of both the unloaded and drug carrier known as CaCO_3_ nanocrystal as seen in the Figure 2. The original crystal natures of loaded CaCO3 nanocrystals are not change or disappear when compared to the unloaded nanocrystals.

### 2.3. Drug Loading and Encapsulation Efficiency

Drug loading and encapsulation efficiency is very important in a drug delivery system; it’s one of the parameters used to evaluate the usability of nanocarriers. As indicated in Table 1, drug loading content has significant effect on the encapsulation efficiency—the higher the drug loading the lower the encapsulation efficiency. The results indicated that the more loading of drug into the CaCO_3_ nanocrystals the higher the percentage of drug lost in the loading medium. This was also observed by other researchers [9,10,11]. The formulation used in this analysis has a loading content of 4.8 and encapsulation efficiency of 97%. Reports from previous data have shown the ability of CaCO_3_ either in the nano form or as microparticles to load drug, or other macromolecules due to the porous nature or nanopores on the surface of the particles, as indicated in the FESEM image in Figure 2, the CaCO_3_ nano/microparticles prepared in the presence of polysaccharides have a large number of nanopores, which provide a strong capability to load drugs regardless of their surface charge and hydrophilicity since the drugs could be loaded into the hybrid nano/microparticles due to the capillary force [12,13].

**Figure 1:**
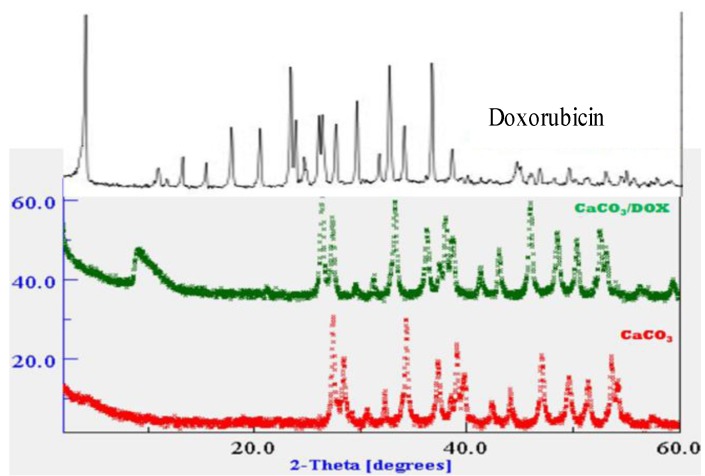
XRD spectra for free doxorubicin, CaCO_3_ nanocrystals and CaCO_3_/DOX nanocrystals.

**Table 1:** Drug loading concentrations, loading content and encapsulation efficiency.

Samples	Weight of nanocrystals (mg)	Weight of drug (mg)	Loading content (%)	Encapsulation efficiency (%)
CaCO_3_ (1)	50	1	4.5	97
CaCO_3_ (2)	50	2	8.9	86
CaCO_3_ (3)	50	3	11.7	75

### 2.4. Elemental Analysis of CaCO_3_/Dox

**Figure 3:**
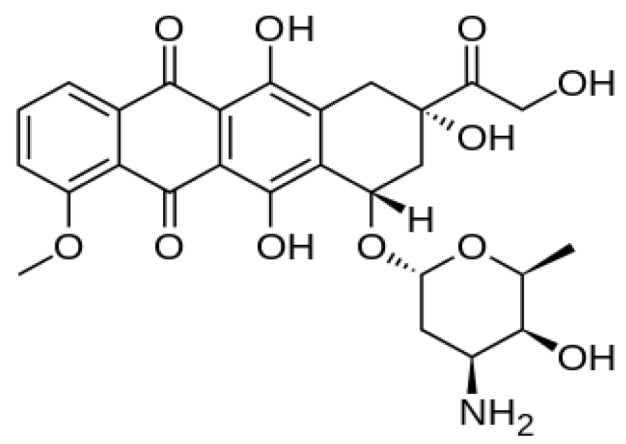
Chemical structure of doxorubicin (C_27_H_29_NO_11_).

The elemental composition of the CaCO_3_/Dox nanocrystal formulation was analysed and is presented in Table 2. The results show the presence of nitrogen from the amine group (NH_2_) and hydrogen from the hydroxyl (OH) group, these elements are only found in the doxorubicin (C_27_H_29_NO_11_) considering the elemental composition in CaCO_3_ which only contains C, O and Ca, therefore, the presence of nitrogen and hydrogen in the CaCO_3_/Dox nanocrystal formulation are clearly from the doxorubicin, as indicated in the formula C_27_H_29_NO_11_ and also shown by the chemical structure in Figure 3. The elementals analysis shown in Table 2 confirmed the intercalation or encapsulation of doxorubicin into the calcium carbonate nanocrystals.

**Table 2:** Elemental composition of drug-loaded calcium carbonate nanocrystals.

Samples	% C	% H	% N	N/H
CaCO_3_/Dox	13.128	0.5954	0.5447	1.09
Doxorubicin	53.91	5.21	2.22	2.34

### 2.5. Doxorubicin Release Profile

The stability of doxorubicin may be affect by the different pH environment (pH dependent) probably due to the increase in solubility of doxorubicin at mildly acidic pH values [12]. In this study pH 4.8 and 7.4 are selected to simulate the extracellular and lysosomal environment, respectively.

**Figure 4:**
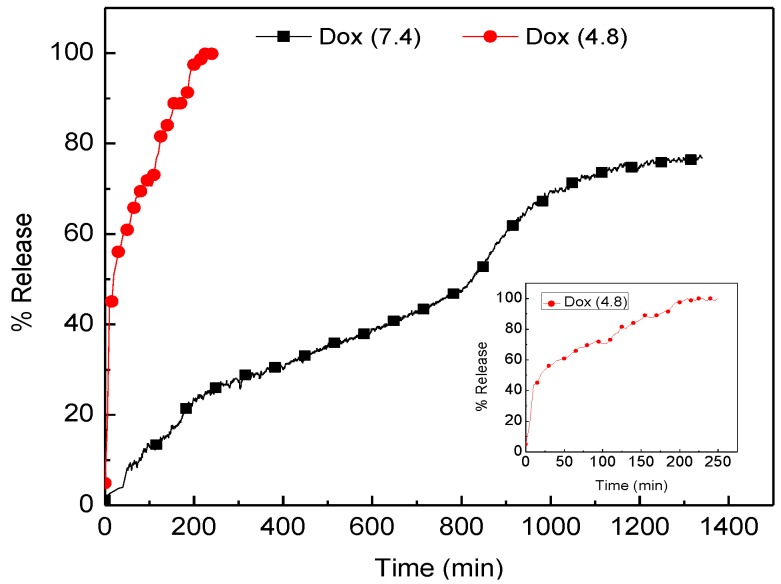
Doxorubicin Release Profiles in PBS, pH 4.8 and 7.4.

The *in vitro* release of doxorubicin from the loaded CaCO_3_ nanocrystals is shown in Figure 4, where the profile of Dox released indicates two different phases of release at pH 4.8, First, is the burst or fast release of 45% of the Dox within 15 min and secondly is the sustained released of Dox for 250 min where approximately 100% of Dox was released. The rate of Dox released at pH 7.4 is notably higher compared to pH 4.8. The cumulative percentage of doxorubicin released at pH 7.4 was approximately 80% within 1,300 min but when the pH of the released medium was changed to pH 4.8 the corresponding release of 80% occurred within 50 min.

The results observed from the release studies were similar to those reported by others researchers [3,13,14,15,16]. It was observed by Gillies *et al.* [16] that a higher drug loading content in inorganic hybrids may result in an elevated amount of drug being released because the diffusion driving force of the concentration gradient is enhanced and the therapeutic efficacy will also be enhanced, since therapeutic effectiveness is closely related to the release of drug from the carrier system. Therefore the slow release of Dox from calcium carbonate nanocrystals at normal blood pH or normal physiological pH is an indication of excellent anticancer drug carrier properties in view of the fact that most of the conventional methods of chemotherapeutic delivery fail to achieved therapeutic concentrations of the drugs needed at the target site or do not have specificity and selectivity for the target cells or tissues, thereby affecting the normal or healthy cells in the body. The excellent property of our nanocrystals is that the Dox released from the calcium carbonate nanocrystals is hardly released in normal tissues or healthy cells (pH = 7.4), while at (pH = 4.8) the Dox may responsively release in tumor tissues, or even within cancer cells, to selectively kill the cancer cells.

### 2.6. CaCO_3_ Nanocrystal Biocompatibility Assay

**Figure 5:**
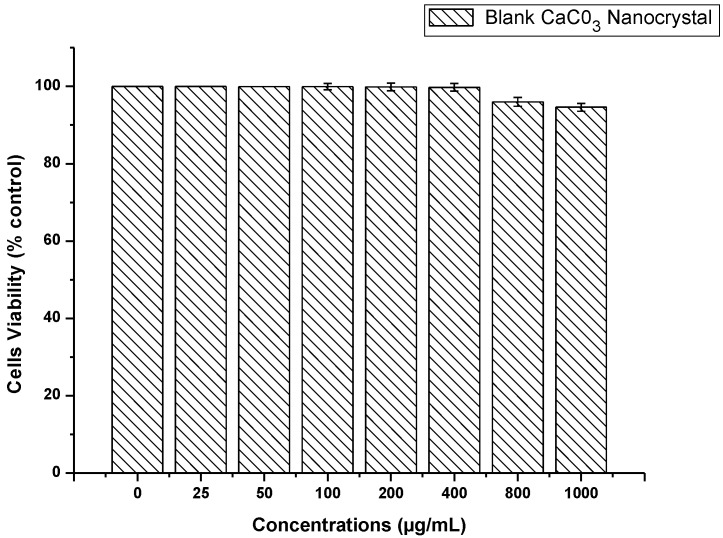
Cytotoxity of blank CaCO_3_ on MG 63 bone cancer cells line.

The effect of blank CaCO_3_ nanocrystals on osteosarcoma bone cancer cell (MG 63) was examined to determine the toxicity of the delivery system for the compatibility studies in biological systems. The data presented in Figure 5, represent the MTT assay results. MMT (3-[4,5-dimethylthiazol-2-yl]-2, 5-diphenyltetrazolium bromide) which is a water-soluble tetrazolium salt, and the assay is based on the principle that mitochondrial dehydrogenase of intact cells converts the soluble yellow MTT tetrazolium salt into an insoluble purple formazan through the cleavage of the tetrazolium ring; the formazan product is impermeable to cell membranes with loss of integrity and therefore only accumulates in healthy cells.

The analysed results revealed a good cytocompatibility of calcium carbonate nanocrystals which showed no apparent toxicity to MG 63 cells, even at higher concentrations of 800 to 1,000 µg/mL, and the viability percentaged were above 90%. Toxicity studies of any nanomaterial are the first evaluation step in cellular interaction and may lead to the next cellular response. This study based on CaCO_3_ nanocrystal cytocompatibility may suggest it as a potential material for biomedical applications, especially as a carrier in drug delivery systems. Calcium carbonate nanomaterials are now one of the leading inorganic materials used in biomedical applications. Abundant research has shown its success in biomedical applications, especially in the delivery of different types of drugs, DNA and other macromolecules, either in combined state as composites or alone [12,13,14].

### 2.7. Doxorubicin and CaCO_3_/Dox MTT Cytotoxicity Assay

**Figure 6:**
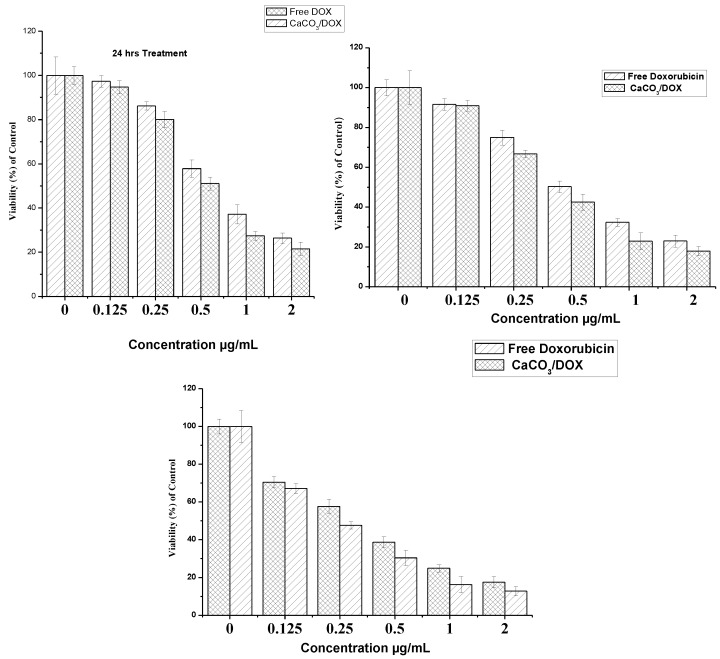
*In-vitro* cytotoxicity study of MG 63 Osteosarcoma cells after 24, 48 and 72 h of exposure to free DOX and the CaCO_3_/DOX nanocrystals.

Cytotoxicity studies of free doxorubicin and Dox encapsulated in calcium carbonate were evaluated on the MG 63 osteosarcoma cell line using an MTT assay. MG 63 cells was treated with different concentrations of free Dox and CaCO_3_/Dox nanocrystals and incubated for periods of 24, 48 and 72 h. It was observed that the cell inhibition rate is dependent on the increase in concentration and incubation periods. From Figure 6, MG 63 was more sensitive to free doxrubicin compared to CaCO_3_/Dox for 24 and 48 h while at 72 h it was observed that CaCO_3_/Dox was found to be more sensitive than free Dox within the expirimental period.

These differences in cytotoxicity are explained by many researchers as result of the mechanism of cellular uptake of the drug. The cellular uptake of free DOX occurs through a passive diffusion mechanism whereby it may be trapped at the P-gap junction and thereby affect the normal cells while in the case of CaCO_3_/DOX, the drug has to be released in a time dependent manner from the CaCO_3_ nanocrystals before exerts its effects on the cells, therefore as the time increases the rate of drug release increases and the concentration also increases, inhibiting the cell growth in a time dependent manner. The cellular uptake of CaCO_3_/DOX nanocrystals was through non-specific endocytosis which may lead to reduced effect of cytosolic free DOX for the P-gp pumping action; this mechanism of CaCO_3_/DOX nanocrystal delivery to tumours may circumvent the effect of multidrug resistant proteins which are always present in cancer cells [16,17]. P-glycoprotein has the capacity of recognising drug and expelling them from the cancer cells only when such drugs are in the plasma membrane, and not when the drug is located in the cytoplasm of the lysosomes after endocytosis [18]. The results indicated a higher therapeutic index of the drug-loaded nanocrystals.

### 2.8. BrdU Cell Proliferation Assay

**Figure 7:**
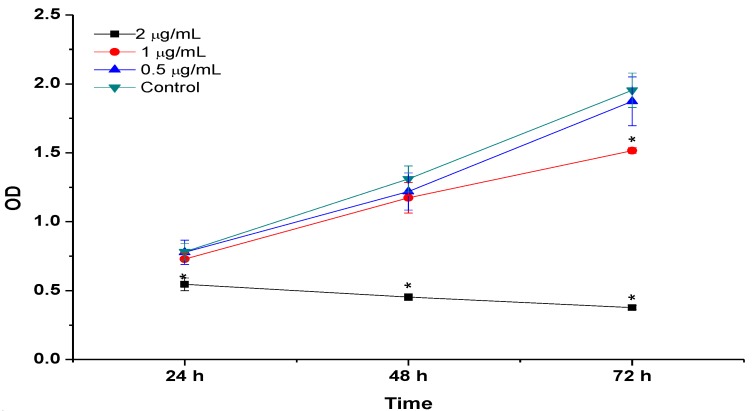
BrdU Cell proliferation assays, MG 63cells was treated with CaCO_3_/Dox for 24, 48 and 72 h incubation period.

Cell proliferation assay was analysed by BrdU for the incubation periods of 24, 48 and 72 h with concentrations of CaCO_3_/DOX ranging from 0 (control) to 2 µg/mL, as shown in Figure 7. The results indicate significantly suppressed MG 63 proliferation in a dose and time dependent manner compared to control or untreated cells. The BrdU proliferation assay results were similar to the MTT ones, confirming the decrease in cell number as indicated in both Figure 5 and Figure 6 respectively.

### 2.9. LDH Release (Membrane Integrity Assay)

LDH releases from the cell are only possible in the cell with the membrane damage, since in normal cells the cytosolic enzymes can leak into extracellular fluids only when the membrane integrity is loses by the cells. LDH release is one of the popular assays used to analyse the cytotoxicity of certain compound or drugs.

**Figure 8:**
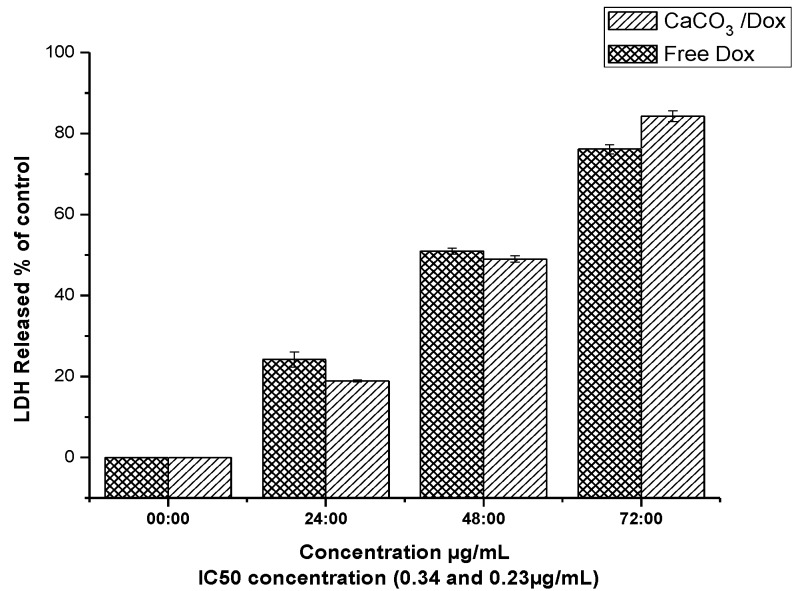
LDH released analysed for membrane integrity assay.

To further investigate the possible cytotoxicity effect of the free Dox and CaCO_3_/Dox, lactate dehydrogenase (LDH) levels were analysed to study the membrane integrity of MG 63 cells after exposure to the IC_50_ concentration (0.34 and 0.23 µg/mL) for 24 h for both free Dox and CaCO_3_/Dox nanocrystals, respectively, for the period of 72 h as indicated in Figure 8. From the analysis an increase in LDH released was observed in all the incubation periods of 24, 48 and 72 h, and the release is also in time dependent, with the highest value being seen at 72 h. Free Dox has the highest LDH release compared to CaCO_3_/Dox nanocrystals at 24 and 48 h, but suddenly at 72 h higher release was recorded for CaCO_3_/Dox nanocrystals compared to free Dox. The gradual increment of LDH release at 72 h for CaCO_3_/Dox may be attributed to the release mechanism of the two treatment, since it was reported that cellular uptake of free Dox is through passive diffusion while that of CaCO_3_/Dox is through specific endocytosis [12,17]. In the case of CaCO_3_/Dox the drug has to be released slowly from the CaCO_3_ nanocrystals before it can exert an effect on the cancer cells, but free Dox has an immediate effect to the cells since it has direct contact to the cells initially.

### 2.10. Morphological Observations

**Figure 9:**
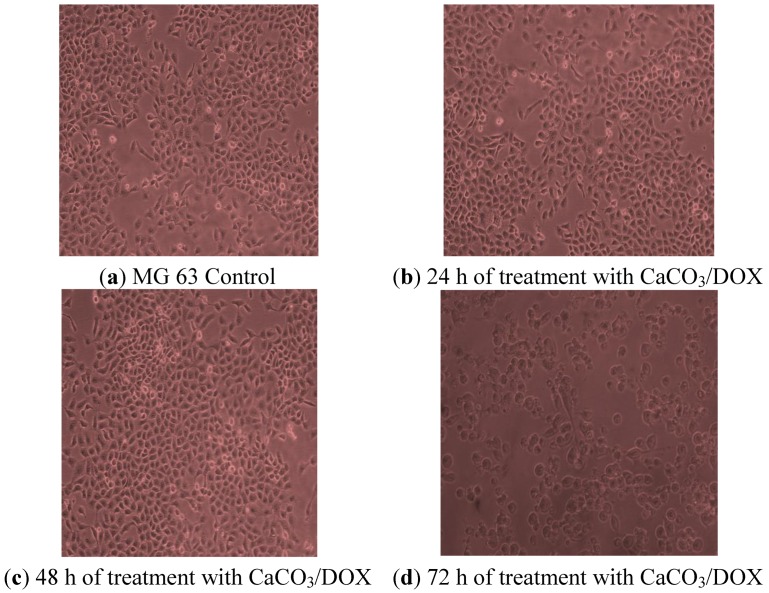
Image of MG 63 cells treated with various for 24, 48 and 72h of incubation period.

MG 63 osteosarcoma cell line was treated with CaCO_3_/DOX nanocrystals for a period of 24, 48 and 72 h, and the morphological observations (Figure 9) revealed that after 24 h of treatment some cells become rounded and detached from the surface of the flask. At 48 and 72 h the majority of the treated cells were rounded and many were suspended in the culture media, while others showed a blabbing characteristic which was visible. The number of treated cells was reduced as compared to the control. Therefore, this study showed effective delivery of anticancer drug and inhibition of cell growth by the loaded calcium carbonate nanocrystals.

### 2.11. Mechanism of Cellular Uptake of Doxorubicin

Doxorubicin hydrochloride is a water soluble anthracycline drug with inherent fluorescence properties and is one of the most common therapeutic drugs used as a research tool in most cancers [19]. Moreover, the fluorescent nature of the Dox molecule can serve to assess the uptake and the imaging of organs or cells can provide accurate information on drug biodistribution [19].

**Figure 10:**
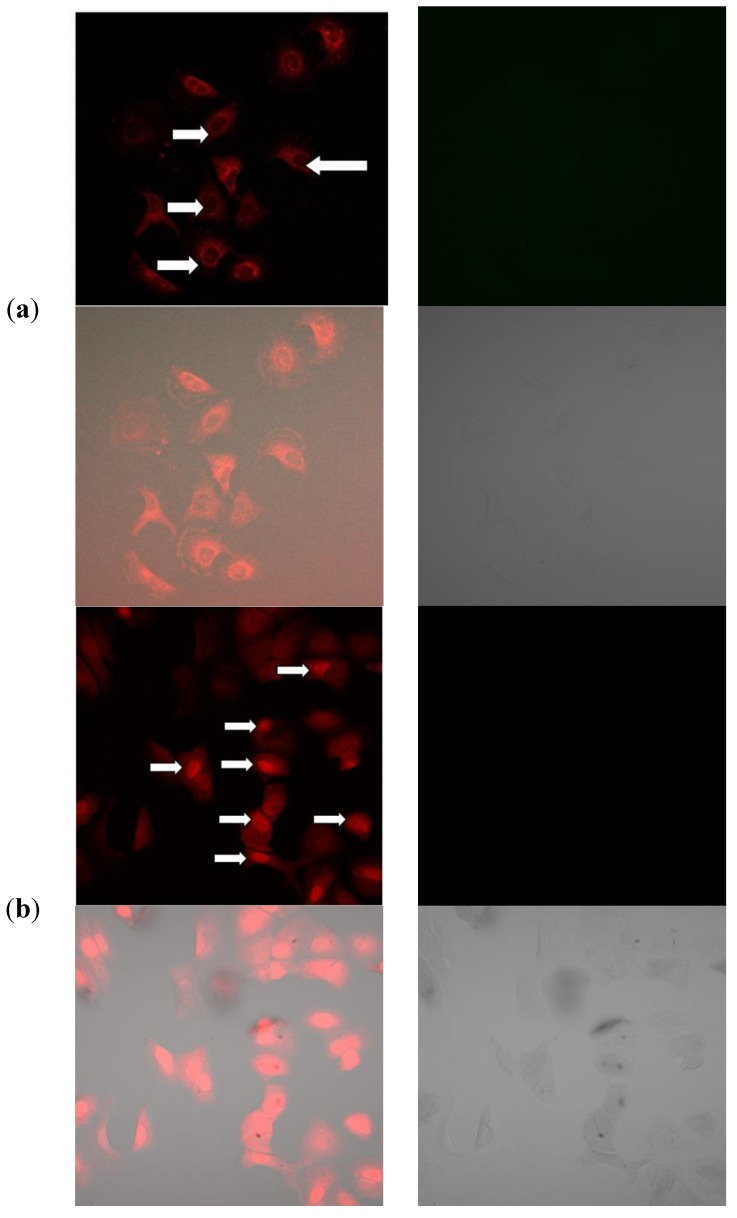
(**a**) Confocal microscopy images of doxorubicin-loaded CaCO_3_ nanocrystal uptake by MG 63 cells, Images are overlapped. Bright field image; (**b**) confocal microscopy images of free doxorubicin uptake by MG 63 cells. Images are overlapped Bright field image.

The differences for the intensity of doxorubicin fluorescence uptake between the cytoplasm and the nuclei are indicated in Figure 10a. This may be as a result of trapping the Dox in the endosomal compartment after cellular uptake before reaching the nucleus [20] and the rate of release of Dox from the CaCO_3_ nanocrystals. Similar research was conducted by Liu *et al.* [21], who observed that the strong fluorescence in the cell cytoplasm was initially released from the nanocrystals during the incubation periods, then diffused from the cytoplasm to the nucleus where it will intercalate in the DNA in the cells, However the diffusion of Dox from the cytoplasm depends on the time taken during the incubation. Thus, the incubation period may increase the red fluorescence from the cytoplasm to the nuclei as observed in Figure 10a, such an uptake mechanism of Dox released by CaCO_3_ nanocrystals occurs through non-specific endocytosis as described by many researchers. However, in the case of free Dox, a strong red fluorescence was observed within the nucleus with less in the cytoplasm, as shown in Figure 10b, The explanation of this trend may lay with the fact that free DOX has immediate direct contact with the MG 63 cells, which may initially induce a substantial inhibition of cell growth that diminishes with increasing time. Also, tumour uptake of free DOX takes place through passive diffusion, which may result in trapping of the drug at the P-gap junction and adverse effects in normal cells [20,21]. This clearly showed a direct contact of the Dox to the cells which was highly concentrated in the nucleus of the cells. This mode of Dox delivery is among the shortcomings of conventional chemotherapy where it shows no specificity and selectivity to cancerous cells.

The data from the confocal images reveals the ability of CaCO_3_ nanocrystals to deliver the doxorubicin into the cellular compartment and also offers evidence of the Dox mechanism of internalisation by the cell through endocytosis. A parallel experiment conducted by different researchers reported differences in the mechanism of cellular uptake between free doxorubicin and the dox delivered by nanoparticles. The same explanation was offered, *i.e*., that the Dox delivered by the nanoparticles is initially trapped in the endosomal compartment with a strong red fluorescence and weaker within the nuclei of the cells [20,21,22,23,24]. Fast release of the total drug by the CaCO_3_ nanocrystals may result in DOX molecules being embedded in DNA and inhibiting the synthesis of nucleic acids. Therefore increasing the amount of DOX entering the cancerous cells in a short period of time will lead to an enhancement of the drug effciency [25].

**Figure 11:**
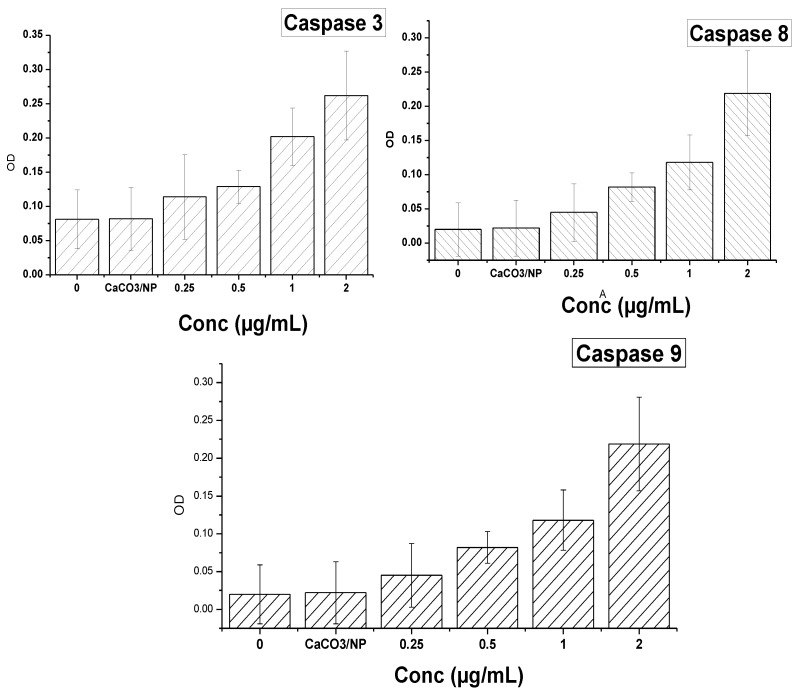
The effect of CaCO_3_/Dox nanocrystals on the levels of caspase-3, 8 and caspase-9 in MG 63 cells.

**Figure 12:**
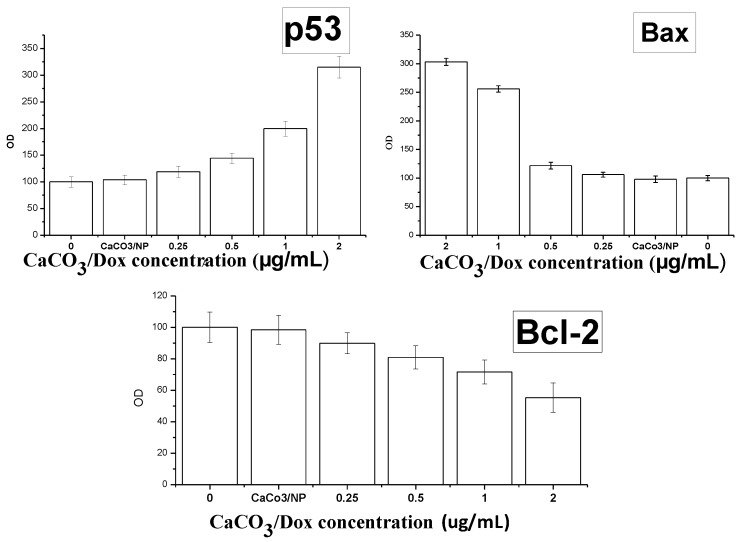
Up-regulation of p53 and Bax and down-regulation of Bcl-2 through the effect of CaCO_3_/Dox nanocrystals on MG 63 cells.

Figure 11 and Figure 12 demonstrate the results of genes and protein expression involved in the cell death and signalling pathway in the MG 63 osteosarcoma cell line after treatment with CaCO_3_/Dox nanocrystals at different concentrations. As shown from the figures, delivery of Dox via calcium carbonate nanocrystals induced apoptosis by up-regulating the p53 gene, Bax, caspases 3, 8 and 9 while the antiapoptotic (Bcl-2) defense protein was down-regulated. Expression of apoptotic caspases 3, 8 and 9 and up-regulation of p53 gene by calcium carbonate nanocrystals loaded with Dox lead to the up-regulation of the pro-apoptotic member of the Bcl-2 family known as Bax, and the expression of these pro-apoptotic and anti-apoptic protein are observed to be concentration dependent as indicated in both Figure 11 and Figure 12. It was previously reported by Salim and Hindi [26] that activation of Bax by up-regulating caspases and tumor suppression gene p53 may induce permeabilization of the outer mitochondrial membrane, which releases soluble proteins from the intermembrane space into the cytosol, where they promote caspases activation [27,28]. To balance between the pro-apoptotic and antiapoptotic defences and death mechanism, a ratio was calculated, and in both the analysed p53, Bax/Bcl-2 and caspases which were concentration dependent, the ratio was > 2-fold higher compared to the untreated or control cells. It was reported that induction of apoptosis by increasing the cell death signalling pathway led to the over-expression of caspase 9 and cleavage of procaspase 9 to form active caspase 9. The active caspase 9 (apoptosis initiator) triggered the cascade of caspase-apoptosis executors, including caspase 3 [12].

Therefore, the analysed data indicate that delivery of Dox through calcium carbonate nanocrystals showed the mechanisms of MG 63 osteosarcoma bone cancer cells death by induction of apoptosis via the up-regulation of p53, Bax and caspases 3, 8 and 9 and down-regulation of the Bcl-2 protein.

## 3. Experimental

### 3.1. Synthesis and Drug Loaded Calcium Carbonate Nanocrystals.

Synthesis of calcium carbonate nanocrystals were carried out in oil-in-water (O/W) microemulsions using a higher pressure homogeniser (HPH) as described in [7]. In this technique, the particle sizes are reduced after leaving the homogenising gap by cavitations, particle collisions, and shear forces. Drug loaded calcium carbonate nanocrystals were prepared by adding an aqueous solution of doxorubicin hydrochloride (1 mg/mL) into the calcium carbonate nanocrystal suspension (containing 20 mg of nanocrystals). The encapsulation of Dox in CaCO_3_ nanocrystals was achieved by continuous stirring of the suspension mixture in the dark overnight at room temperature. Calcium carbonate nanocrystals containing Dox were washed, centrifuged and oven dried (FD 115, Fisher Scientific, Limburg, Germany) at 55 °C.

### 3.2. Characterisation of Calcium Carbonate Nanocrystals

The following instruments were employed for observation and characterisation of calcium carbonate nanocrystals. The morphology and particle size of the nanocrystals were analysed by transmission electron microscopy (TEM, Hitachi H-7100, Hitachi, Kyoto, Japan) and field emission scanning electron microscopy (FESEM, JEOL 7600F, JEOL, München, Germany) GmbH. The crystal powder was characterized by X-ray powder diffraction (XRD, λ = 0.15406 nm A) on a Rigaku x-ray diffractometer (Rigaku, Tokyo, Japan) with Cu Kα radiation at a scan rate of 4 °/min.

### 3.3. Determination of Drug Loading Content and Encapsulation Efficiency

The drug loading and encapsulation efficiency in the CaCO_3_ nanocrystals were analysed by calculating the difference between the total of the drug fed (Wt) and the free drug (Wf) concentrations in the nanoparticles supernatant per weight of CaCO_3_ nanocrystals. The formulated mixture was centrifuged at 5,000 g/15 min and the amount of free DOX remaining in the supernatant of solution was determined by measuring the absorbance at 485 nm on a UV-vis spectrophotometer (PerkinElmer Lambda 35, Perkin Elmer, Boston, MA, USA). Data were given as average measurements of three independent values. Drug loading and encapsulation efficiency are calculated according to [8] as stated in Equations (1) and (2) below:

(1) $$\text{Loading content}=\frac{w_{t}\;-w_{f}}{w_{nP}}\;X100$$

where Wt = the total weight of drug fed, Wf = the weight of non-encapsulated free drug, Wnp = the weight of the nanoparticles.

(2) $$\text{Encapsulation efficiency}=\;\frac{w_{t}-w_{f}}{w_{t}}\;X100$$

where Wt = the total weight of drug fed, Wf = the weight of non-encapsulated free drug.

### 3.4. *In Vitro* Controlled Drug Release Study

To measure the *in vitro* release of drug, drug-loaded calcium carbonate nanocrystal suspensions (containing approximately 10 mg of nanoparticles) in 50 mL of PBS buffer (pH 4.8 and 7.4) were prepared. At predetermined intervals, the drug concentration was determined by measuring the absorbance at a selected wavelength λ_max_ = 480 on a UV-vis spectrophotometer. The data are given as mean ± standard deviation (SD) based on the measurements of the samples from three replicates.

### 3.5. *In Vitro* Evaluation of Cytotoxicity

MG 63 Osteosarcoma cancer cell line was purchased from the American Type Culture Collection (ATCC, Manassas, VA, USA), and cultured in DMEM medium supplemented with 10% fetal bovine serum, L-glutamine and 15 mmol/L penicillin 100 U/mL, and streptomycin 100 µg/mL. The culture was then incubated in 5% CO at 37 °C. Cells at 80%–90% confluence were used for seeding and treatment.

### 3.6. Cells Seeding and Treatment

The cells were seeded into a 96-well plate at a density of 5 × 10^4^ per well and incubated for 24 h. The cells were co-cultured with different concentrations of DOX and CaCO_3_/DOX nanocrystal suspension (0 to 2 µg/mL) for periods of 24, 48 and 72 h. After the exposure was completed the media was aspirated and washed with PBS before being replaced with another media without serum prior to MTT treatment. MTT reagent (Sigma Aldrich, St. Louis, MO, USA) 20 µL in PBS was added into each well and the plate was incubated at 37 °C for 4 h (Thermo Fisher Scientific LPG, Hudson, NH, USA). The culture medium in the wells was removed and 200 µL of dimethylsulfoxide (DMSO) was added into the wells to release the color product into the solution. The plate was kept in the dark room for 30 min and optical density of the solution was measured at 570 nm with a microplate reader. The experiment was conducted in triplicate.

### 3.7. LDH Release Membrane Integrity Assays

Cells were seeded into a 96-well plate containing a seeding density of 5 × 10^4^ and incubated for 24 h. The medium was removed and replaced with CaCO_3_/Dox nanocrystal suspensions using IC_50_ concentrations (0.34 and 0.23 µg/mL for both free Dox and CaCO_3_/Dox respectively). The plate was incubated at 37 °C for 24, 48 and 72 h. After the incubation, 2 μL of lysis buffer was added into the positive control wells, and the plate was centrifuged (Thermo Fisher Scientific LPG) at 1,500 rpm for 10 min at 37 °C. After centrifugation, 50 μL of a membrane integrity assay reagent was added into the wells. The plates were incubated for 10 min at 37 °C protected from light. Thirty (30 µL) of HCl 1N stop reagent was added into the wells and the fluorescence of the samples was measured at 560 nm (excitation) and 590 nm (emission) on the microplate reader. The percentage of cytotoxicity was calculated with respect to the positive control wells whereby the lysed cells were assumed to have 100% lactate dehyrogenase [LDH] release.

### 3.8. BrdU Proliferation Assay

Cell proliferation was analyzed based on the incorporation of BrdU (5-bromo-2′-deoxyuridine) into the synthesized DNA during cell proliferation. MG 63 cells were seeded in 96-well plates at a density of 5 × 10^3^ cells per well and incubated for 24 h, the cells were washed with PBS and then treated with various concentrations of CaCO_3_/Dox, control was treated with 0.1% DMSO, after treatment the cells was subjected to a 5-bromo-2-deoxyuridine labeling assay according to the manufacturer protocols (Roche Diagnostics GmbH, Mannheim, Germany). The absorbance was measured at 550 nm using a microplate reader and the data was presented as mean ± S.D.

### 3.9. Morphological Examination

Microscopic observation is one of the more reliable tests for determining and quantifying cell viability and cell death. MG 63 cells were treated with various concentration of CaCO_3_/DOX for 24, 48 and 72 h. After 72 h, the cells were viewed under an inverted light microscope. Apoptotic characteristics were identified by the appearance of cell shrinkage, nuclear condensation, and/or the presence of membrane-bound apoptotic bodies

### 3.10. DOX Cell Uptake and Drug Release Investigations

MG 63 osteosarcoma cells were seeded into a 6-well plate and allowed to grow for 3 days in 100% culture medium (DMEM). On reaching 80% cell confluence, the medium was replaced with 1 mL of fresh culture medium supplemented with 25 mM HEPES containing CaCO_3_/DOX nanocrystals and incubated at 37 °C for 6 h. The cells were washed three times with PBS before analysis by confocal microscopy. Fluorescence images were recorded using the auto-fluorescence of DOX excited at 488 nm. The fluorescence emission (between 565 and 630 nm) was visualised by confocal microscopy (Olympus Confocal Microscope System, Tokyo, Japan).

### 3.11. Elemental Analysis Determination

Two (2 mg) of both CaCO_3_ nanocrystals and pure doxorubicin were accurately weighed in a tin capsule and weighed twice once after filling the tin capsule and again after folding. The prepared samples are delivered by a rotating autosampler into a combustion furnace with a pulse of pure oxygen. All combustible materials in the sample are combusted to gases, *i.e.*, all carbon-bearing materials are converted into CO_2_ and nitrogen-bearing materials to N_2_.The resulting combustion gases (product) are separated by GC column and measured by a thermal conductivity detector (TCD).

### 3.12. Measurement of Caspase-3 and Caspase-8 and 9 Activities

Proteolytic activities of caspase 3, 8 and 9 were measured by colorimetric assay kits (Sigma-Aldrich) according to the manufacturer’s instructions. Briefly, cells were seeded in a dish at a seeding density of 1 × 10^6^ and after reaching confluence, serum-containing medium was aspirated and co-cultured with various concentrations of CaCO_3_/Dox, in a serum-free medium for 24 h. The cell pellets were suspended in a cell lysis buffer and incubated on ice for 10 min. The lysate was vortexed every 15 min. After centrifugation at 11,000 *g* for 15 min, supernatants were collected and for caspase 3, 8 and 9 activity measurement 20 µL of cell lysate were added immediately to a buffer containing a *p-*nitroaniline (pNA)-conjugated substrate for caspase-3 (Ac-DEVDpNA), -8 (Ac-IETD-pNA) and (LEHD-pNlabeled) for caspase 9. The samples were incubated for 1 h at 37 °C. The fluorescence was detected in a microplate reader (SpectraFluor, TECAN, Sunrise, Austria). The concentration of the released pNA was calculated from the absorbance values at 405 nm and caspase activities are expressed as fold increases over the non-treated cell control.

### 3.13. Enzyme-Linked Immunosorbent Assay (ELISA)

Bcl-2/Bax and activity of p53 gene were evaluated. The cells were seeded at a density of 1 × 10^6^ per dish and incubated overnight at 37 °C, then the cells were treated with various concentrations of CaCO_3_/Dox and incubated for 72 h, total protein was extracted from the cells, using lysis buffer-containing protease inhibitor cocktail set III and phosphatase inhibitor cocktail set I (Calbiochem, EMD Biosciences, San Diego, CA, USA). The protein concentrations of all the samples were determined with A Bicinchoninic Acid Protein Assay Kit (Invitrogen Carlsbad, CA, USA). The levels of Bcl-2 and Bax proteins were quantified using the Human Bcl-2/Bax ELISA Kit, according to the manufacturer’s instructions (Human Apoptosis regulator Bcl-2/bax; Cusabio, CA, USA).

## 4. Conclusions

A biobased calcium carbonate nanocrystal with the capacity of carrying anticancer drugs and effectively deliver them to target cells was developed. The calcium carbonate nanocrystals with encapsulated Dox significantly inhibited MG 63 cells with efficient and sustained release of DOX with slow release at normal physiological pH 7.4 and a faster release rate in an acidic environment at pH 4.8 was observed. The synthesized delivery carrier system may offer a successful and promising potential application for many therapeutic agents with more confidence in doxorubicin for the clinical treatment of osteosarcoma bone cancer.
